# From Anxiety to Hardiness: The Role of Self-Efficacy in Spanish CCU Nurses in the COVID-19 Pandemic

**DOI:** 10.3390/medicina60020215

**Published:** 2024-01-26

**Authors:** Fernanda Gil-Almagro, Fernando José García-Hedrera, Francisco Javier Carmona-Monge, Cecilia Peñacoba-Puente

**Affiliations:** 1 Departamento de Psicología, Facultad de Ciencias de la Salud, Universidad Rey Juan Carlos, 28922 Alcorcón, Spain; fgilalmagro@gmail.com; 2 Hospital Universitario Fundación Alcorcón, 28922 Alcorcón, Spain; fjgarciah@gmail.com; 3 Hospital Universitario Santiago de Compostela, 15706 A Coruña, Spain; javichun@gmail.com

**Keywords:** self efficacy, anxiety, critical care nursing, hardiness

## Abstract

*Background and Objectives*: Evidence shows that throughout the COVID-19 pandemic, nurses suffered from emotional symptoms, yet in spite of this, few studies within “positive psychology” have analyzed the emergence/promotion of positive traits, such as hardiness. In this context, the present study aimed to test a model regarding the mediating role of self-efficacy between anxiety experienced at the beginning of the COVID-19 pandemic and hardiness assessed six months later among nurses in critical care units (CCU) in Spain. *Materials and Methods*: An observational, descriptive, prospective longitudinal study with two data collection periods: (1) from the 1 to the 21 June 2020 (final phase of the state of alarm declared in Spain on 14 March) in which socio-demographic and occupational variables, anxiety (Depression, Anxiety and Stress Scale, DASS-21), self-efficacy (General Self-Efficacy Scale, GSES) and basal resilience (Resilience Scale-14, RS-14) were assessed, and (2) a follow-up 6 months later (January–March 2021) in which hardiness (Occupational Hardiness Questionnaire, OHQ) was evaluated. To analyze the data, multivariate regressions were performed using the PROCESS macro (simple mediation, model 4). *Results*: A total of 131 Spanish nurses from CCUs, with a mean age of 40.54 years (88.5% women) participated in the study. Moderate and severe levels of anxiety were observed in 19.1% of the sample. Significant and positive correlations were observed between self-efficacy, hardiness and resilience (all *p* < 0.001). Significant negative correlations were observed between anxiety and self-efficacy (*p* < 0.001), hardiness (*p* = 0.027) and resilience (*p* = 0.005). The indirect effect of anxiety on hardiness through self-efficacy was significant (Effect (SE) = −0.275 (0.100); LLCI = −0.487, ULCI = −0.097), contributing to 28% of the variance, including resilience (*p* = 0.015), age (*p* = 0.784), gender (*p* = 0.294) and years of experience (*p* = 0.652) as covariates. A total mediation was observed (non-significant anxiety-hardiness direct effect; Effect (SE) = −0.053 (0.215), *t* = 0.248, *p* = 0.804, LLCI = −0.372, ULCI = 0.479). *Conclusions*: The results suggest that in Spanish CCU nurses, anxiety experienced at the beginning of the COVID-19 pandemic may contribute to the development of hardiness through positive resources such as self-efficacy.

## 1. Introduction

Previous research has pointed out the psychoemotional consequences experienced by nurses in Critical Care Units (CCU) as a consequence of the COVID-19 [1,2,3] pandemic. These consequences have been associated with several risk factors such as fear of contagion, caring for infectious patients, working in specialized units such as the CCU [4,5], or the fact of being a mother and a nurse in the CCU [6]. A recent meta-analysis in nurses reported prevalent rates of stress, anxiety and depression of 43%, 37% and 35%, respectively [7]. Specifically, using the Depression Anxiety Stress Scale (DASS-21), moderate to extremely severe levels of depression, anxiety and stress have been found in 22–29% of CCU nurses [8]. Despite the importance of the above data, there has been little research into the possible positive consequences that the pandemic may have had on nurses within the framework of “positive psychology”, such as the effect on the promotion of hardiness through coping with adverse circumstances [9,10].

Hardiness has been studied, within the general framework of “resilience factors (RF)” [11], as a positive consequence after experiencing situations of a certain traumatic intensity, understood as a tendency to perceive life differently [12]. Specifically, its authors refer to three dimensions: commitment (capacity to be involved in what one is doing and to consider any initiative that is carried out as important), control (belief in one’s capacity to influence events and to manage one’s own life), and challenge (considering changes as an opportunity for progress, rather than a threat) [12].

Research into hardiness began by focusing on a minority coping style in a specific traumatic situation (i.e., concentration camps), and subsequent studies have focused more on its possible consequences than on its antecedents. In relation to this, it has been observed that hardiness is associated with lower levels of stress and fewer health problems, less emotional exhaustion, anxiety, depression and somatization [13,14]. Specifically, in CCU nurses, hardiness has been studied as a protective health factor, showing that hardy CCU nurses cope effectively and adaptively with stress and the uncertain context of these specific units, and consequently, in the long term, are less likely to leave the unit prematurely [15]. It has also been shown that nurses’ hardiness can help increase work engagement and improve job performance when faced with stressful circumstances in the workplace [16].

The definition of hardiness refers to a series of attitudes and actions that facilitate the transformation of stressful situations into opportunities for growth, through the promotion of courage and motivation [12]. In this context, the importance of coping, social interaction and self-care have been raised [12,17]. One of the variables that can be considered as promoting hardiness is self-efficacy [18]. Although most of the research is correlational in nature, significant positive relationships have been observed between self-efficacy and hardiness [19,20] and some studies in stressful situations have shown that the dispositional effect of hardiness is partly due to an indirect effect of contextual self-efficacy beliefs [21]. Specifically, in a sample of intensive care and emergency professionals, a clear relationship between hardiness and self-efficacy has been observed in predicting psychological distress [22].

Given the highly stressful situation experienced during the COVID-19 pandemic by CCU nurses [23], taking into account the definition of self-efficacy [16], it is of particular interest to study its role as a possible factor promoting hardiness. In this context, one might ask whether in CCU nurses, their perception of self-efficacy might contribute to the anxiety experienced at the onset of the COVID-19 pandemic generating hardiness in the mid-term. It is known that self-efficacy plays an important role after psychological trauma, contributing to reducing short- and long-term anxiety levels through a positive evaluation of one’s ability to cope with the traumatic event and its consequences [24]. During the COVID-19 pandemic, self-efficacy has been studied in nurses as a personality trait that mitigates levels of anxiety, depression and burnout generated by pandemic-derived trauma [25,26].

**Figure 1:**
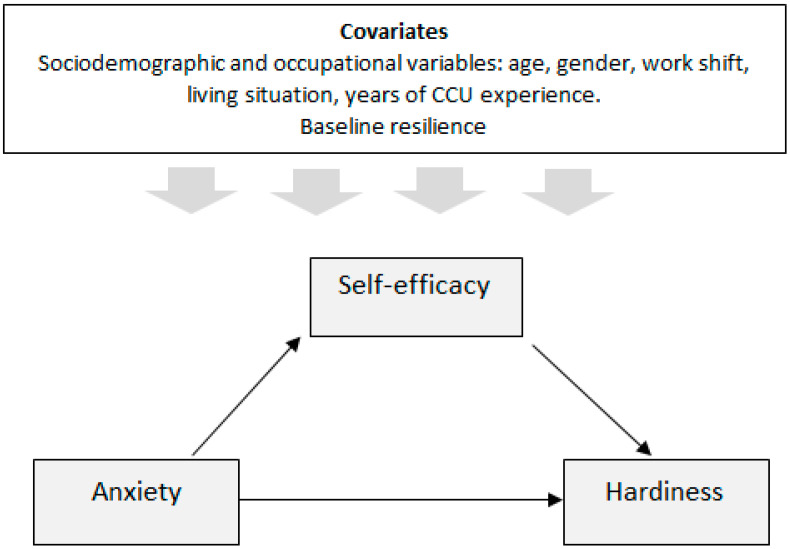
Conceptual model proposed: from anxiety to hardiness with self-efficacy as a mediator.

From this perspective, taking into account the scarce research carried out from “positive psychology”, it may be of interest to associate self-efficacy and hardiness as protective personality traits in CCU nurses, and as possible strengths for coping with the anxiety derived from the COVID-19 pandemic. To our knowledge, there are no studies analyzing the generation of hardiness through self-efficacy, within the context of the COVID-19 pandemic as a traumatic situation, using a prospective design. Specifically, the aim of the present study has been to analyze the mediating role of self-efficacy between the anxiety experienced at the beginning of the COVID-19 pandemic and the hardiness assessed six months later among CCU nurses in Spain. To this end, a mediation model will be proposed (see Figure 1). Specifically, the following hypothesis was proposed: (H1) A full mediating role of self-efficacy would be observed between anxiety (at the beginning of the COVID-19 pandemic) and hardiness (6 months later), i.e., self-efficacy is a necessary variable in the association of anxiety with hardiness. This mediation would be significant considering the inclusion of socio-demographic and occupational variables and baseline levels of resilience.

## 2. Materials and Methods

Design: An observational, descriptive, longitudinal prospective study was carried out with two periods of data collection in relation to the COVID-19 pandemic: (1) between 1 and 21 June 2020 (the final phase of the state of alarm declared in Spain on 14 March) and (2) a follow-up 6 months after the state of alarm was finalized (January–March 2021). At the first time point, levels of anxiety and self-efficacy were assessed, as well as certain sociodemographic and occupational data and baseline levels of resilience, as possible covariates to be controlled for in the model. At the second time point, hardiness was assessed.

**Figure 2:**
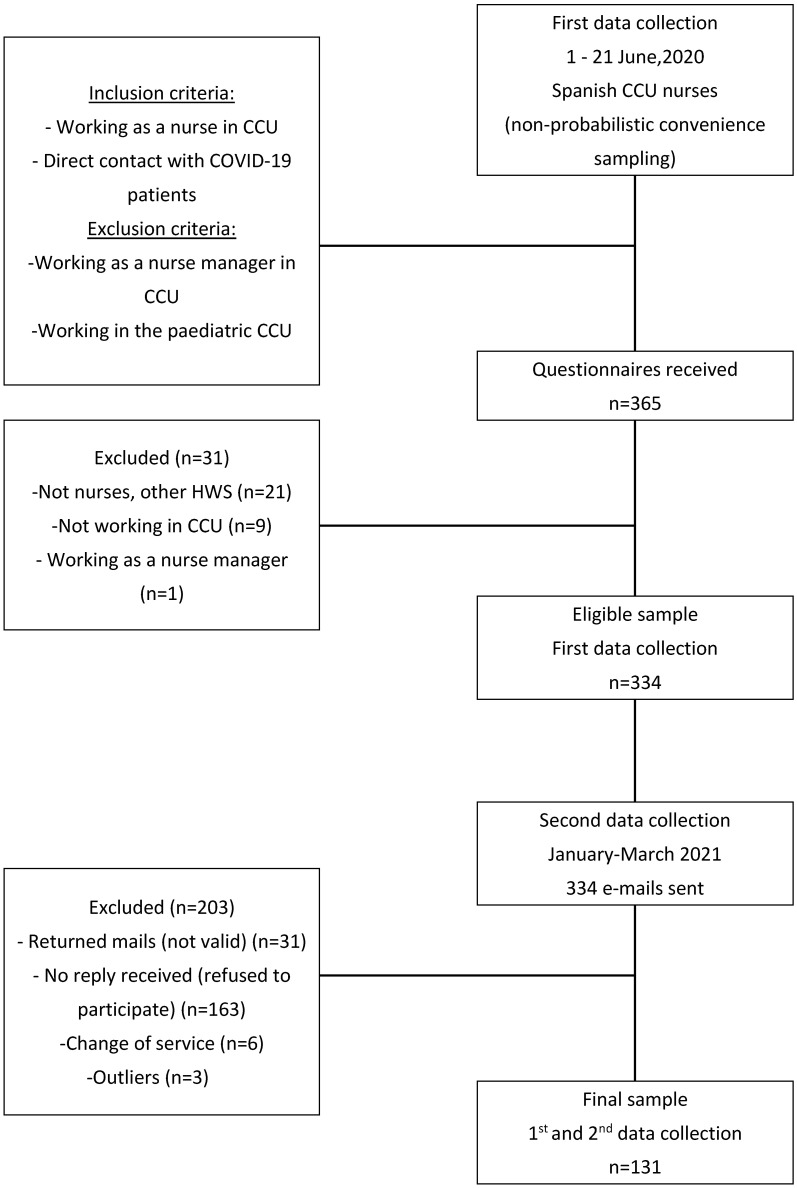
Graphical presentation of inclusion and exclusion criteria.

Participants: The sample was composed of nurses from critical care units in Spain in contact with COVID-19 patients. The sample was selected using non-probabilistic convenience sampling. A minimum sample size of *n* = 120 was considered for prospective mediation studies [27]. The following inclusion criteria were taken into account: working as a nurse in the CCU during the data collection period and in direct contact with COVID-19 patients. The following exclusion criteria were considered: change of service during the study period, working as a nurse manager in the CCU, or working in the pediatric CCU (due to the absence of COVID-19 in infants and newborns). Aware of the usual sample loss in longitudinal studies with this population, in addition to the contextual conditions of data collection (COVID-19 pandemic) [28,29], a minimum sample size of 300 participants was established at the first time point, resulting in a sample of 334 participants in that first period of data collection (between 1 and 21 June 2020). Six months later, in the data collection of the second time point (January–March 2021), 131 nurses out of the 334 maintained their participation, therefore constituting the final sample of this study. Figure 2 shows a graphical presentation of inclusion and exclusion criteria. It should be noted that no statistically significant differences were observed in the variables of interest (anxiety, self-efficacy and baseline resilience) between the participants who did not complete the two phases of the study (participating only in the first time point, *n* = 203) and those who completed the study (*n* = 131).

Procedure: Data collection was performed by an online electronic questionnaire (google forms) designed for this purpose by the research team. The objective of the study was presented at the beginning of the questionnaire, and informed consent was requested to use the data of the participants in the study. Missing values were monitored through the design of the online questionnaire itself, where all questions had to be completed in order to progress through the questionnaire. The link was sent to CCU nurses in the Spanish health system who had been in contact with patients affected by COVID-19, and was also disseminated through social networks (Facebook, twitter, LinkedIn and WhatsApp), corporate e-mails from public and private health services in the Spanish system were also used. Data collection occurred between the 1 and 21 June 2020. For the dissemination of the assessment protocol at the second time point, the e-mails of the CCU nurses who finally participated in the first time point were used, thus requesting, once again, their participation in this second phase of the study. This second data collection was carried out from January 2021 to March 2021.

### 2.1. Variables and Data Collection

#### 2.1.1. Outcome Variables

Anxiety: The Spanish version [30] of the Depression, Anxiety and Stress Scale (DASS-21) [31], designed to evaluate states of depression, anxiety and stress was administered. Each dimension consists of seven items with a Likert-type response format of four alternatives from 0 (“it has not happened to me”) to 3 (“it has happened to me a lot” or “most of the time”). The score for each of the dimensions ranges from 0 to 21 points. For the purpose of the present study only the anxiety dimension was used, showing a Cronbach’s alpha of 0.81 in our sample. Additionally, to establish the different degrees of anxiety, the established classification was followed: normal (from 0 to 7), medium (from 8 to 9), moderate (from 10 to 14), severe (from 15 to 19) and extremely severe (scores over 20) [31].

Self-efficacy: The Spanish version [32] of the General Self-Efficacy Scale (GSES) [33] was used, consisting of 10 items that measure the perception of competence to manage life situations, with a 4-point Likert-type response format between 1 (“not at all true”) and 4 (“completely true”). The total score ranges from 10 to 40; a higher score indicates better levels of self-efficacy. The reliability of the general self-efficacy scale in our study measured by Cronbach’s alpha was 0.86.

Hardiness: We proceeded to the administration of the Occupational Hardiness Questionnaire (OHQ) in its Spanish version [34], composed of 21 items assessing 3 dimensions, commitment, control and challenge, with a Likert-type response format ranging from 1 (“completely disagree”) to 4 (“completely agree”). For this study, the total hardiness score was considered (range from 21 to 84), with higher scores reflecting more hardiness [34]. Cronbach’s alpha in our study was 0.82.

#### 2.1.2. Covariates

Certain sociodemographic and occupational variables, along with baseline resilience, were considered as possible covariates to be considered in the model. Sociodemographic (gender, age, marital status) and occupational variables (transfer from their usual unit to the CCU due to the COVID-19 pandemic, employment situation, degree, years of experience in the CCU, work shift and Autonomous Community to which their hospital belongs) were collected using an ad hoc instrument developed by the research team.

As a baseline measure of resilience, the Resilience Scale (RS-14) [35] in its Spanish version was administered, as it is the most widely used measure within the so-called resilience factors [11]. It consists of 14 items on a 7-point Likert-type response format from 1 (strongly disagree) to 7 (strongly agree), with the total scale score ranging from 14 to 98, so that the higher the score the greater the resilience. Cronbach’s alpha in our study was 0.94.

#### 2.1.3. Data Analysis

The IBM SPSS Statistics version 27.0 (IBM Corp. Armonk, New York, NY, USA) was used for data analysis [36]. Possible outliers (*n* = 3, 2.29%) were identified using the Mahalanobis distance and excluded of the sample.

Descriptive statistics and Cronbach’s alpha analysis were calculated for all variables analyzed. Qualitative variables were described with frequencies (n) and percentages (%) and quantitative variables with mean and standard deviation (SD). Pearson’s correlation was used to analyze the bivariate correlation between variables, and for the association between variables, the χ^2^ test and Student’s t test were used, according to the nature of the variables analyzed. The results were considered statistically significant for values of *p* < 0.05. Different multivariate regressions were carried out using the PROCESS macro, specifically through a simple mediation analysis (model 4). As can be seen in Figure 1, we performed a mediation model, considering self-efficacy (assessed at the first time point) as mediator (i.e., M) of the relationship between anxiety assessed in the same period (i.e., X) and hardiness assessed six months later (i.e., Y). In the mediation analyses, we calculated direct, indirect, and total effects [37]. The effect of X (i.e., anxiety) on Y (i.e., hardiness) was referred to as the direct effect (c’ path). The effect of X on Y via M (i.e., self-efficacy) was referred to as the indirect effect (ab path). Path a represented the effect of X on M, whereas path b was the effect of M on Y when controlling for the effect of X. Lastly, we determined the total effect of X on Y (path c), which is the sum of direct and indirect effects. To test the significance of indirect effects, bias-corrected bootstrap confidence intervals (BC-CI) were calculated [37]. The bootstrap estimates were based on 5000 bootstrap samples [38]. A 95% CI was used. Significant effects were those in which the confidence interval did not contain zero. Mediation analyses were conducted using the PROCESS macro for SPSS version 27.0 [36,38].

### 2.2. Ethical Considerations

The study was approved by the Ethics and clinical research committee of the hospital (Reference: 2088) and all participants signed the informed consent form before starting the questionnaire. The current study was supported by the Spanish Society for Intensive Nursing and Coronary Units (SEEIUC). It was conducted in accordance with the national and international guidelines of the code of ethics, the declaration of Helsinki and the code of good practice and SAS Order 3470/2009. The processing of the patients’ personal data which were collected in this study complied with Organic Law 15/1999 of 13 December on the Protection of Personal Data (LOPD) and with Regulation (EU) No. 2016/679 of the European Parliament and of the Council of 27 April 2016 on Data Protection (GDPR).

## 3. Results

### 3.1. Sample Characteristics

A total of 131 Spanish CCU nurses participated. As can be seen in Table 1, almost the totality of the sample was composed of women. The mean age of the participants was 40.54 years, with a minimum age of 22 and a maximum age of 70, most of them were married. Regarding the employment status, more than half of them were permanent employees with rotating shifts. The average number of years of work experience in the CCU was 11.76, ranging from new entrants to 35 years.

### 3.2. Descriptive Statistics and Correlations between Variables

As can be seen in Table 2, mean anxiety scores (6.10) were at the borderline of the first tercile of the theoretical range (0–17). Considering the classification established for the scale in relation to anxiety levels, the data show the following distribution in our sample: 66.4% (*n* = 87) normal levels, 4.5% (*n* = 19) medium levels, 16% (*n* = 21) moderate levels, 3.1 (*n* = 4) severe levels. No participant showed extremely severe levels. The mean values for self-efficacy and hardiness were considered moderately high. Baseline resilience scores were considered high.

Correlation analyses (see Table 3) showed significant relationships between all variables. Negative correlations were observed between anxiety and self-efficacy (*p* < 0.001), hardiness (*p* = 0.027) and resilience (*p* = 0.005). The correlation between anxiety and hardiness had a small strength of association. Positive correlations were observed between self-efficacy and hardiness, self-efficacy and resilience, and hardiness and resilience (all *p* < 0.001).

### 3.3. Relationship of Anxiety, Self-Efficacy and Hardiness with Socio-Demographic and Employment Variables: Covariate Analysis

Table 4 shows the relationships of the variables under study (anxiety, self-efficacy, hardiness and basal resilience) with age, gender, cohabitation status (with a partner vs. without a partner), work shift (permanent vs. non-permanent), and years of experience in the CCU. Results showed statistically significant relationships between age and anxiety (r = −0.273, *p* = 0.002; CI [−0.420, −0.103]) and between years of CCU experience and anxiety (r = −0.173, *p* = 0.048; CI [−0.331, 0.002]). Statistically significant differences were also observed between men and women in experienced anxiety (*t* = −2.444, *p* = 0.016; CI [5.67–7.14]). No statistically significant differences were observed for the rest of the variables analyzed. Therefore, in the proposed mediation model, gender, age and years of experience in the CCU are proposed as covariates to be considered. Baseline resilience was also included as a covariate.

### 3.4. Mediation Analysis: Prediction of Hardiness Based on Anxiety as an Antecedent and Proposing Self-Efficacy as a Mediator

**Figure 3:**
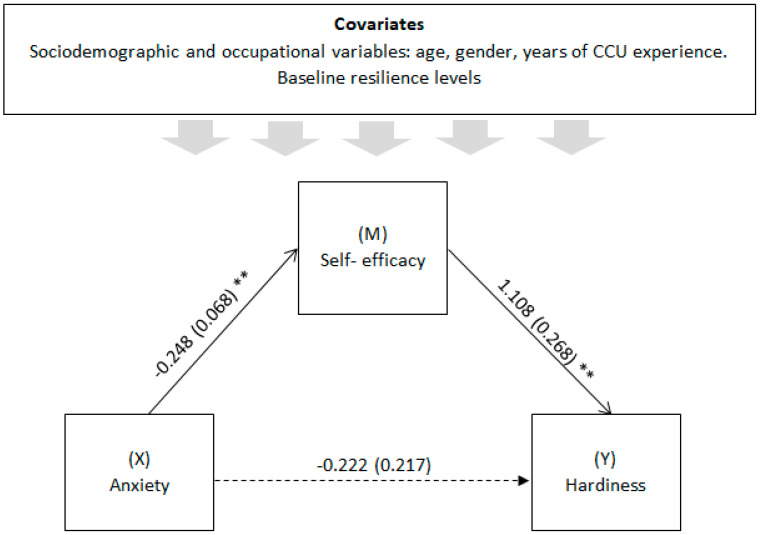
Path diagram illustrating the direct and mediated effects pathways linking anxiety to hardiness with self-efficacy as a mediator. Notes: Simple mediation analysis with anxiety as an independent variable, hardiness as a dependent variable and self-efficacy as a mediator. Values are unstandardized regression coefficients (SE in parentheses) and associated *p*-values (** *p* < 0.01). Association in parentheses = direct effect (controlling for indirect effects). Solid lines indicate significant pathways and dashed lines indicate non-significant pathways.

Table 5 shows the regression analyses carried out on the prediction of hardiness through anxiety experienced six months earlier, using self-efficacy as a mediating variable. Figure 3 shows the model with the coefficients found.

As can be seen in Table 5, once the possible effect of the covariates (gender, age, years of experience in the CCU and baseline resilience) were controlled for, the mediation results showed that the model explains 28% of the variance of hardiness six months after COVID-19 confinement (F = 8.269, *p* < 0.001). The effect of anxiety on hardiness was entirely mediated by self-efficacy, with the direct anxiety-hardiness personality effect being non-significant (*p* = 0.308). These results confirm the hypothesis proposed (H1).

## 4. Discussion

The main aim of the present study has been to test a model that includes hardiness as an outcome in CCU nurses, considering the anxiety experienced during the COVID-19 pandemic as the antecedent and self-efficacy as the mediating variable through the use of a prospective study (establishing the first time point as the final phase of the state of alarm declared in Spain on 14 March, and the second time point 6 months later).

Taking into account the widely documented [1,2,3,7,8] anxiety experienced by healthcare workers and the derived psychopathology, we aimed to expand the focus on the development of certain personality factors within the framework of “positive psychology”. This approach is especially relevant within the healthcare community, since it allows us to delve into the protective factors of their psychoemotional health in the face of highly stressful stimuli (as in the case of the anxiety experienced by the COVID-19 pandemic), contributing to their psychological well-being [39,40].

The data found regarding the anxiety experienced by CCU nurses in the first stage of the pandemic reflect the emotional consequences in this group, which is widely documented in previous research [1,2,3,7,8]. Specifically, in our sample, we found that 19.1% (*n* = 25) presented moderate to extremely severe anxiety. These percentages are lower than those found in a recent meta-analysis [8] using the same tool (DASS-21), which indicated percentages of 28.6% for moderate to extremely severe anxiety. It should be taken into account that this meta-analysis was carried out on CCU health professionals and not only on nurses. Other studies carried out in China [41] have established percentages of 12.3% of moderate to extremely severe anxiety, which is similar to our results. The variability in the data depends on issues such as the specific health care population assessed, the number of participants, cultural differences or the exact moment of measurement. A striking aspect in this sense is the finding of Greenberg et al. [42] who found lower levels of anxiety in CCU health professionals compared to those in non-CCU during the pandemic, justifying these results based on the higher workloads inherent to the position itself, previous experience in this highly stressful environment and greater training of this group during the pandemic.

Regarding self-efficacy, studies carried out on nurses with the same instrument during the pandemic indicated scores very similar to those found in our research [43,44]. With regards to hardiness, as noted, our data showed moderately high scores. To the best of our knowledge, there are no studies assessing this variable in nurses during the pandemic, although its evaluation has been carried out in other populations, yielding equally moderate scores [45,46,47], although these did not use the Spanish version [34] administered in our study.

The analyses carried out showed that anxious symptoms correlate negatively with self-efficacy at the first time point and with hardiness six months later, although in the latter the strength of association was weaker. Previous studies carried out on nurses during the pandemic have similarly stated that self-efficacy correlates negatively with anxiety [48]. Taking this into account, the importance of focusing on or reducing anxiety seems to be justified, given that this disorder also represents the biggest occupational health problem within nursing, after musculoskeletal disorders [49], highlighting the need to implement therapies such as mindfulness to mitigate nurses’ anxiety [49]. Previous research in nursing indicates that psychological preparation for a disaster favors self-efficacy [50], whilst self-efficacy, in turn, contributes to optimism [51]. Thus, it would be of interest to address not only the high emotional impact of the pandemic on CCU nurses, or the stressful situations derived from their highly specialized work [23,52,53], but also the ability to manage anxiety through self-efficacy to increase the possibility of generating hardiness as a consequence.

As noted, hardiness is associated with people’s health, specifically contributing to resilience in high-stress situations [54,55]. Although there are other factors, such as social support, that can also influence resilience, previous research suggests that hardiness is the main “internal” factor contributing to resilience [56]. Thus, research studies carried out in different populations indicate that hardiness acts as a moderator between stressors linked to COVID-19 and anxiety and depression [45]. This holds true for the relationship between mindfulness and emotional exhaustion (considered a key factor in burnout syndrome) [46], being associated with higher scores of uncertainty tolerance [47]. Previous literature reveals correlations between hardiness, stress and happiness in nurses, constituting a protective factor against stress and a facilitating factor for happiness [57].

The prospective design used in our study is of special interest. The self-efficacy measured in the first phase of the pandemic (during the confinement period) showed a significant relationship with hardiness assessed 6 months later. This ability of self-efficacy to relate to hardiness in the mid-term is especially relevant if we keep in mind that hardiness has been shown to be a protective trait against burnout in the nursing community [58]. This result reinforces the uniqueness of so-called positive psychology, which points out the importance of independently analyzing psychological variables that contribute to well-being, in our case those related to self-efficacy. The study of both approaches (psychoemotional illness and positive psychology) is complementary and independent, since it has been observed that the variables that predict illness are not the same as those that predict well-being [59].

Thus, the results obtained showed that, when the anxiety levels associated with the pandemic were higher, the more relevant it became to have self-efficacy to promote the development of a hardiness. A particularly interesting fact about the proposed model is that a direct relationship between anxiety and hardiness was not observed six months later, thus showing that the relationship between anxiety and hardiness must be mediated (in its entirety) by self-efficacy to be significant. In this sense, self-efficacy is a fundamental variable to strengthen the link between anxiety and hardiness during the experience of stressful work situations by CCU nurses. Previous literature has shown that self-efficacy plays a mediating role between optimism and happiness, which in turn influences coping with difficult situations, since people with low self-efficacy show pessimistic attitudes in response to problematic circumstances [25].

Another relevant finding of the model derives from controlling for covariates (the model is significant, including age, gender, years of experience in the CCU and basal resilience). The model provides an explanation for 28% of the variance in hardiness. Although the percentage of variance is significant, it would be of interest to include other variables such as social support, self-esteem, cognitive fusion, emotional regulation and gratitude, which have also been found to be relevant variables in adaptive coping during the COVID-19 pandemic [60,61,62,63].

In short, the results of this study provide data of interest for the promotion of hardiness in adverse situations such as the COVID-19 pandemic in CCU nursing professionals. Hardiness has demonstrated its importance for the psychoemotional health of healthcare professionals [64], and its role as a protective trait against burnout has also been observed in nurses [58]. Given the relevance of this trait, our model shows the possibility of managing the anxiety developed by CCU nurses after stressful situations through self-efficacy, favoring the possibility of developing hardiness. At an applied level, after reflecting on the results obtained, it seems essential to develop emotional intervention strategies focused not only on reducing emotional symptoms but also on developing protective traits to promote hardiness, such as self-efficacy.

The data from this study highlights once again [65,66], in response to a health crisis such as COVID-19, the need to address the mental health of our healthcare professionals, particularly nursing professionals in CCUs [42]. This attention, as has been pointed out, should be dynamic, flexible, adaptable to the evolution of the pandemic and include technology in its different phases [67]. Furthermore, in view of our results and previous research, the gender perspective should be incorporated [66,68,69] and evaluation instruments should be used to assess the clinical evolution of health professionals throughout the entire crisis.

There is no doubt that the COVID-19 pandemic has contributed to raising awareness of the need to address the mental health of health professionals. A systematic review on psychological interventions for healthcare professionals during the COVID-19 pandemic [70] includes a total of 10 interventions from different approaches such as psychoeducation, cognitive-behavioral therapy or acceptance and commitment therapy, six of them using new technologies (phone, web applications or video). These interventions assess the effects on different emotional symptoms including depression, anxiety, and stress during the pandemic. This review highlights the effectiveness of these interventions, pointing out the need for their maintenance over time and their implementation by qualified professionals in the workplace.

In the Spanish health system, although, in general terms, interventions aimed at the mental health of professionals have not been carried out, different studies conducted in Spain have shown that implementing this type of intervention is not only necessary but also possible, and positive results have been obtained [66,71,72]. A brief psychological intervention, based on anxiety management, emotional regulation, and value oriented-behavior, was shown to be effective in reducing emotional symptoms in professionals [66]. In another study, through the design of a specific application (PsyCovidApp) that included interventions on emotional skills, healthy lifestyle behaviors, burnout, and social support, showed positive effects on depression, anxiety, and stress [72]. Another brief intervention [71] (five-week, two-hour group sessions) aimed at promoting emotional regulation skills for coping with stressful situations showed good acceptability and preliminary efficacy in reducing the emotional impact of the pandemic on nursing staff [71].

In this context, our data support the need for the psychological care of health professionals, especially in situations of high stress and anxiety, such as the COVID-19 pandemic. As an additional contribution, our research emphasizes the role of self-efficacy. Self-efficacy has been shown to be a variable of interest in reducing emotional symptoms in the aforementioned interventions [72,73]. Our study proposes the implementation of psychological interventions on self-efficacy to promote healthy characteristics, within positive psychology, such as hardiness. In this context, a recent investigation among CCU nurses caring for COVID-19 patients has highlighted the importance of clinical competence (including skills, perceptions and emotions) in psychological empowerment [74]. Self-efficacy constitutes an essential element of clinical competence in healthcare professionals [75]. Although psychological interventions, in this sense, from positive psychology, are not abundant, initial research provides promising results. As an example, a recent study assessed the effects of a brief intervention from positive psychology on the mental health of nursing staff in German hospitals, observing positive short-term results on reflection and promotion of self-management skills, ultimately improving clinical competence [76]. Our data suggest the need for psychological intervention protocols in this direction that incorporate the assessment of hardiness as the ultimate goal of the intervention.

### Limitations

The present study has a number of limitations that should be taken into account in the interpretation of the results. On the one hand, the study was carried out using a convenience sample of Spanish CCU nurses, which affects the generalizability of the results. Therefore, the data is not representative of the complete population (nurses) of Spain. Moreover, it cannot be assured that the study participants are representative of CCU nurses in Spain, especially if we also take into account that in Spain there is no specialism (at an educational level) for CCU nurses. On the other hand, the important sample loss between the first and second time point is noteworthy. This sample loss has been found in these types of studies, especially in Health Care Workers (HCWs), and during the COVID-19 pandemic [28,29]. Moreover, as far as the present study is concerned, it is worth recalling the absence of differences in the outcome variables between the participants who did not complete the study (participating only in the first time point, *n* = 203) and those who completed the study (*n* = 131). The use of self-report questionnaires for the measurement of anxious symptoms may be considered a bias, although it is the most common and established form of assessment with adequate indicators of validity and reliability depending on the instrument used. Finally, it would have been of interest to take into account the emotional symptoms of the participants prior to the COVID-19 pandemic in order to include it as a covariate.

## 5. Conclusions

The present study, through a prospective observational design, highlights the role of self-efficacy as an intermediate variable in the generation of hardiness from the anxiety experienced at the onset of the COVID-19 pandemic in Spanish CCU nurses. A particularly relevant fact is that the model proposed (anxiety-self-efficacy-hardiness) requires the action of self-efficacy for the generation of hardiness. Despite the limitations of the study, the model contributes to the explanation of 28% of the variance in hardiness, after controlling for baseline resilience and certain socio-demographic and occupational variables. Within these variables, it is necessary to take into account gender, age and years in the profession, as our data, in line with previous research, show that being female, being young and having less work experience are associated with higher levels of anxiety.

Given that hardiness is considered a protective trait for the mental health of HCWs, the results point to the need to promote the development of self-efficacy for adaptive anxiety management in the sample and circumstances of the present study. We consider future research along these lines to be necessary in order to go deeper into the issues addressed by the present study.

## Figures and Tables

**Table 1:** Descriptive statistics of sociodemographic and occupational variables (*n* = 131).

		*n*	%	Mean	SD
Age (in years)				40.54	10.02
Experience CCU				11.76	9.34
Gender	Male	15	11.45		
	Female	116	88.50		
Transfer to CCU ^1^	Yes	29	22.10		
	No	102	77.86		
Family status	Married	88	67.18		
	Single	34	25.95		
	Separated	9	6.87		
Education level ^2^	Bachelor’s or equivalent (level 6)	88	67.20		
	Master’s or equivalent (level 7)	38	29		
	Doctorate or equivalent (level 8)	5	3.8		
Employment	Permanent contract	78	59.50		
status	Interim	28	21.40		
	Temporary contract	25	19.10		
Work shift	Rotational	57	43.50		
	Greater than 10 h	47	35.90		
	Fixed shift M/A/N ^3^	22	16.80		
	Shift 12 h/Wards	5	3.80		

SD: Standard deviation. ^1^ Being transferred from your home unit to the ICU. ^2^ Organizing information on education according to The International Standard Classification of Education (ISCED, 2011); https://uis.unesco.org/en/topic/international-standard-classification-education-isced, accessed on 9 January 2024. ^3^ Fixed morning shift. Fixed afternoon shift or fixed night shift.

**Table 2:** Descriptive statistics of anxiety, self-efficacy, hardiness and resilience (*n* = 131).

	Mean	SD	95% CI	Median	IQR	Sample Range	Asymmetry	Kurtosis	Cronbach’s Alpha
Anxiety	6.10	3.95	5.42–6.79	6	5	0–17	0.507	−0.273	0.81
Self-efficacy	29.20	3.33	28.62–29.78	30	3	20–40	−0.017	1.955	0.86
Hardiness	66.52	9.60	64.86–68.18	65	13	27–84	−0.512	1.338	0.82
Resilience	78.03	14.39	75.54–80.51	81	14	14–98	−1.611	3.504	0.94

SD: Standard Deviation; CI: Confidence Intervals; IQR: Interquartile range.

**Table 3:** Correlation analyses between anxiety, self-efficacy, hardiness and resilience (*n* = 131).

	Anxiety	Self-Efficacy	Hardiness	Resilience
Anxiety	1	−0.387 (*p* < 0.001)	−0.193 (*p* = 0.027)	−0.242 (*p* = 0.005)
Self-efficacy		1	0.495 (*p* < 0.001)	0.504 (*p* < 0.001)
Hardiness			1	0.408 (*p* < 0.001)
Resilience				1

**Table 4:** Covariate analysis of the relationship of anxiety, self-efficacy and hardiness with socio-demographic and employment variables.

		Anxiety	Self-Efficacy	Hardiness	Resilience
Age		R^2^ = −0.273 *p* = 0.002	R^2^ = 0.035 *p* = 0.696	R^2^ = 0.015 *p* = 0.864	R^2^ = −0.020 *p* = 0.820
Gender	woman (*n* = 116)	Mean = 6.40 (SD = 3.99)	Mean = 29.11 (SD = 3.35)	Mean = 66.15 (SD = 9.62)	Mean = 78 (SD = 13.94)
	man (*n* = 15)	Mean = 3.80 (SD = 2.80) *p* = 0.016	Mean = 29.93 (SD = 3.23) *p* = 0.372	Mean = 69.40 (SD = 9.21) *p* = 0.218	Mean = 78.26 (SD = 18.09) *p* = 0.946
Work shift	permanent (*n* = 22)	Mean = 6.13 (SD = 3.24)	Mean = 29.50 (SD = 3)	Mean = 66.31 (SD = 7.44)	Mean = 80.63 (SD = 11.61)
	non-permanent (*n* = 108)	Mean = 6.08 (SD = 4.11) *p* = 0.955	Mean = 29.21 (SD = 3.35) *p* = 0.711	Mean = 66.68 (SD = 9.98) *p* = 0.844	Mean = 77.89 (SD = 14.40) *p* = 0.402
cohabitation status	with a partner (*n* = 88)	Mean = 5.85 (SD = 3.77)	Mean = 29.52 (SD = 3.07)	Mean = 66.53 (SD = 9.75)	Mean = 78.42 (SD = 15.41)
	without a partner (*n* = 43)	Mean = 6.62 (SD = 4.30) *p* = 0.294	Mean = 28.55 (SD = 3.76) *p* = 0.121	Mean = 66.51 (SD = 9.38) *p* = 0.990	Mean = 77.23 (SD = 12.16) *p* = 0.659
years of experience in the CCU		R^2^ = −0.173 *p* = 0.048	R^2^ = 0.079 *p* = 0.371	R^2^ = 0.066 *p* = 0.457	R^2^ = 0.032 *p* = 0.720

**Table 5:** Simple mediation model. Effects of anxiety (X: antecedent) on hardiness (Y: outcome) through self-efficacy (M: mediator).

Effects of Anxiety on Self-Efficacy (X → M)					
VD: Self-Efficacy (M)	*B (SE)*	*t*	*p*	95% CI	
				Lower	Upper
VI: Anxiety (X)	−0.248 (0.068)	−3.632	<0.001	−0.383	−0.113
Gender (covariate)	−0.219 (0.787)	−0.278	0.780	−1.778	1.339
Age (covariate)	−0.030 (0.036)	−0.856	0.393	−0.101	0.040
Work experience in years (covariate)	0.027 (0.037)	0.716	0.474	−0.047	0.101
Baseline Resilience (covariate)	0.099 (0.017)	5.653	<0.001	0.064	0.134
Model Summary R = 0.578 R^2^ = 0.334 F = 12.469 *p* < 0.001					
**Effects of Anxiety and Self-Efficacy on Hardiness (X + M → Y)**					
**VD: Hardiness (Y)**	***B (SE)***	***t***	***p***	**95% CI**	
				**Lower**	**Lower**
VI: Anxiety (X)	−0.053 (0.215)	0.248	0.804	−0.372	0.479
M: Self-efficacy (M)	1.108 (0.268)	4.12	<0.001	0.577	1.640
Gender (covariate)	−2.477 (2.35)	−1.051	0.294	−7.140	2.185
Age (covariate)	−0.029 (0.107)	−0.273	0.784	−0.242	0.183
Work experience in years (covariate)	0.051 (0.113)	0.451	0.652	−0.172	0.274
Baseline Resilience (covariate)	0.145 (0.059)	2.456	0.015	0.028	0.262
Model Summary R = 0.536 R^2^ = 0.287 F = 8.269 *p* < 0.001					
**Effects of Anxiety on Hardiness (X → Y)**					
**VD: Hardiness (Y)**	***B (SE)***	***t***	***p***	**95% CI**	
				**Lower**	**Lower**
VI: Anxiety (X)	−0.222 (0.217)	−1.022	0.308	−0.652	0.208
Gender (covariate)	−2.721 (2.502)	−1.087	0.279	−7.674	2.232
Age (covariate)	−0.063 (0.114)	−0.557	0.578	−0.289	0.162
Work experience in years (covariate)	00.081 (0.119)	0.675	0.500	−0.156	0.318
Baseline Resilience (covariate)	0.255 (0.056)	4.564	<0.001	0.144	0.366
Model Summary R = 0.434 R^2^ = 0.188 F = 5.766 *p* < 0.001					
Total Effect of X on Y Effect (SE) = −0.222 (0.217) *t* = −1.022 *p* = 0.308 LLCI = −0.652 ULCI = 0.208					
Direct Effect of X on Y Effect (SE) = 0.053 (0.215) *t* = 0.248 *p* = 0.804 LLCI = −0.372 ULCI = 0.479					
Indirect effect of X on Y Effect (SE) = −0.275 (0.100) LLCI = −0.487 ULCI = −0.097					

LLCI, ULCI: Lower limits (LL) and Upper limits (UL) of 95% confidence interval (CI).

## Data Availability

Research data will be available upon request to the corresponding author.
